# Supplementary material for: An intestinal sphingolipid confers intergenerational neuroprotection
Source: Nat Cell Biol. 2023 Aug 3;25(8):1196–207. doi: 10.1038/s41556-023-01195-9 (PMC10415181; doi:10.1038/s41556-023-01195-9)
Supplement: Supplementary file 1 — Reporting Summary [file 41556_2023_1195_MOESM1_ESM.pdf]

nature portfolio

Corresponding author(s): Roger Pocock

Last updated by author(s): 6/14/2023

Reporting Summary

Nature Portfolio wishes to improve the reproducibility of the work that we publish. This form provides structure for consistency and transparency in reporting. For further information on Nature Portfolio policies, see our Editorial Policies and the Editorial Policy Checklist.

Statistics

For all statistical analyses, confirm that the following items are present in the figure legend, table legend, main text, or Methods section.

n/a	Confirmed	
☐	☒	The exact sample size (*n*) for each experimental group/condition, given as a discrete number and unit of measurement
☒	☐	A statement on whether measurements were taken from distinct samples or whether the same sample was measured repeatedly
☐	☒	The statistical test(s) used AND whether they are one- or two-sided<br>*Only common tests should be described solely by name; describe more complex techniques in the Methods section.*
☒	☐	A description of all covariates tested
☐	☒	A description of any assumptions or corrections, such as tests of normality and adjustment for multiple comparisons
☐	☒	A full description of the statistical parameters including central tendency (e.g. means) or other basic estimates (e.g. regression coefficient) AND variation (e.g. standard deviation) or associated estimates of uncertainty (e.g. confidence intervals)
☐	☒	For null hypothesis testing, the test statistic (e.g. *F*, *t*, *r*) with confidence intervals, effect sizes, degrees of freedom and *P* value noted<br>*Give P values as exact values whenever suitable.*
☒	☐	For Bayesian analysis, information on the choice of priors and Markov chain Monte Carlo settings
☒	☐	For hierarchical and complex designs, identification of the appropriate level for tests and full reporting of outcomes
☒	☐	Estimates of effect sizes (e.g. Cohen's *d*, Pearson's *r*), indicating how they were calculated

Our web collection on statistics for biologists contains articles on many of the points above.

Software and code

Policy information about availability of computer code

Data collection	Wormbase (www.wormbase.org, release WS285), Zeiss Zen (v2.0) software, Fiji software (v2.0.0), Wormlab (WL-15) software (MBF Bioscience).
Data analysis	Data analysis was performed using:<br>GraphPad Prism v9.5<br>Integrated Genomics Viewer v2.12.2<br>featureCounts v1.5.2<br>RNAsik pipeline v1.5.0<br>STAR v2.5.2b<br>Picard Markduplicates v2.18.0<br>EdgeR-quasi v3.26.8

For manuscripts utilizing custom algorithms or software that are central to the research but not yet described in published literature, software must be made available to editors and reviewers. We strongly encourage code deposition in a community repository (e.g. GitHub). See the Nature Portfolio guidelines for submitting code & software for further information.

Data

Policy information about availability of data

All manuscripts must include a data availability statement. This statement should provide the following information, where applicable:

- Accession codes, unique identifiers, or web links for publicly available datasets
- A description of any restrictions on data availability
- For clinical datasets or third party data, please ensure that the statement adheres to our policy

RNA sequencing data obtained in this work has been deposited at NCBI under the GEO accession number GSE214425. Previously published ChIP-seq datasets for CEH-60 and PQM-1 TFs that were re-analyzed here and were used to identify binding peaks in the asah-1 promoter using IGV are available under accession codes GSE112981 and GSE25811. All data are available in the main text or the extended data. Source data have been provided in Source Data.

Human research participants

Policy information about studies involving human research participants and Sex and Gender in Research.

Reporting on sex and gender	N/A
Population characteristics	N/A
Recruitment	N/A
Ethics oversight	N/A

Note that full information on the approval of the study protocol must also be provided in the manuscript.

Field-specific reporting

Please select the one below that is the best fit for your research. If you are not sure, read the appropriate sections before making your selection.

☒ Life sciences ☐ Behavioural & social sciences ☐ Ecological, evolutionary & environmental sciences

For a reference copy of the document with all sections, see nature.com/documents/nr-reporting-summary-flat.pdf

Life sciences study design

All studies must disclose on these points even when the disclosure is negative.

Sample size

Sample size was based on previous experiments and prior literature using similar experimental paradigms:

Reference for nervous system analysis:
Neumann, B. & Hilliard, M.A. Loss of MEC-17 leads to microtubule instability and axonal degeneration. Cell Rep 6, 93-103 (2014). doi: 10.1016/j.celrep.2013.12.004.
Norgaard, S., Deng, S., Cao, W. & Pocock, R. Distinct CED-10/Rac1 domains confer context-specific functions in development. PLoS Genet 14, e1007670 (2018). doi: 10.1371/journal.pgen.1007670

Reference for expression analysis:
Rasoul Godini and Roger Pocock. Characterization of the Doublesex/MAB-3 transcription factor DMD-9 in Caenorhabditis elegans. G3 (Bethesda). 2023 Feb 9;13(2):jkac305. doi: 10.1093/g3journal/jkac305.

Data exclusions	No data was excluded.
Replication	All data were generated over multiple days and in triplicate, with all replication attempts successful.
Randomization	Experiments were not randomized and controlling covariates was not necessary. In all experiments, control vs. experimental samples were analyzed in parallel. For functional analysis, groups were allocated based on genotype, confirmed by PCR or based on fluorescent marker expression for rescue strains.
Blinding	Study was blinded unless mutant phenotypes were easily observable.

Reporting for specific materials, systems and methods

We require information from authors about some types of materials, experimental systems and methods used in many studies. Here, indicate whether each material, system or method listed is relevant to your study. If you are not sure if a list item applies to your research, read the appropriate section before selecting a response.

Materials & experimental systems

n/a	Involved in the study
☒	☐ Antibodies
☒	☐ Eukaryotic cell lines
☒	☐ Palaeontology and archaeology
☐	☒ Animals and other organisms
☒	☐ Clinical data
☒	☐ Dual use research of concern

Methods

n/a	Involved in the study
☒	☐ ChIP-seq
☒	☐ Flow cytometry
☒	☐ MRI-based neuroimaging

Animals and other research organisms

Policy information about studies involving animals; ARRIVE guidelines recommended for reporting animal research, and Sex and Gender in Research

Laboratory animals	Caenorhabditis elegans hermaphrodites were used - see attached strain list for full details. The stage of animals imaged/analysed were L4 larvae, 1-day, 2-day or 3-day adults (depending on the experiment). Specific ages are described in the methods and specific Figure legends. Hermaphrodites and males were used to generate new strains via crossing.
Wild animals	The study did not involve wild animals.
Reporting on sex	Hermaphrodites were solely used in our analysis. This is except for analysis of embryos and early larval stages where the sex could not be categorically determined, however, males are 0.1% of the population so extremely rare.
Field-collected samples	This study did not involve samples collected from the field.
Ethics oversight	No ethical guidance was required as this was a C. elegans study.

Note that full information on the approval of the study protocol must also be provided in the manuscript.
